# In Vitro Antioxidant and Prooxidant Activities of Red Raspberry (*Rubus idaeus* L.) Stem Extracts

**DOI:** 10.3390/molecules27134073

**Published:** 2022-06-24

**Authors:** Rasa Garjonyte, Jurga Budiene, Linas Labanauskas, Asta Judzentiene

**Affiliations:** Department of Organic Chemistry, Center for Physical Sciences and Technology, Sauletekio Avenue 3, LT-10257 Vilnius, Lithuania; rasa.garjonyte@ftmc.lt (R.G.); jurga.budiene@ftmc.lt (J.B.); linas.labanauskas@ftmc.lt (L.L.)

**Keywords:** *Rubus idaeus* L., f. Rosaceae Juss, total phenolic content, HPLC/DAD/TOF, amperometry, hydrogen peroxide scavenging, antioxidant/prooxidant activity

## Abstract

Leaves and stems of red raspberry (*Rubus idaeus*) are used in Lithuanian folk medicine. Healing properties of raspberry are related to the content of bioactive compounds, mainly polyphenols. Extracts of raspberry leaves contained higher total phenolic content (TPC) (1290 mg/L, expressed in gallic acid equivalent) compared to that in extracts of stems or peeled bark (up to 420 mg/L and 598 mg/L, respectively). To find out whether the collection time of herbal material was critical for the properties of the extracts, the stems were collected at different times of the year. TPC in the extracts depended more on extraction conditions rather than on the sampling time. Antioxidant activity of raspberry stem and bark extracts tested by spectrophotometric (DPPH^●^ scavenging) and electrochemical (cyclic and differential pulse voltammetry) assays correlated with TPC. DPPH radical scavenging activity values for stem, leaf, and bark extracts were as follows: ≤1.18 ± 0.07, 1.63 ± 0.10, and ≤1.90 ± 0.04 (mmol/L, TROLOX equivalent), respectively. Assessed electrochemically, hydrogen peroxide-scavenging activity of extracts was independent on TPC. The latter activity was related to the presence of some protein in the extract as revealed by gel electrophoresis. Prooxidant activity of raspberry stem extracts was dependent on solution pH and temperature.

## 1. Introduction

Red raspberry (*Rubus idaeus* L., f. Rosaceae Juss.) is a shrub that grows wild in temperate countries. Edible berries of the plant (*Rubi idaei fructus*) contain numerous bioactive substances: minerals (potassium, phosphorus, magnesium, calcium, iron, etc.), vitamins, anthocyanins, ellagitannins, and various other polyphenolic compounds [1,2]. Both berries and green leaves or young shoots of the plant traditionally are used in folk medicine to treat common cold, fever, or ailments of gastrointestinal tract, to relieve menstrual cramps and to stimulate labor [3,4,5]. Chemical composition of raspberry leaf extracts and their bioactive properties such as antioxidant, anti-inflammatory, antimicrobial, diaphoretic, cytotoxic, cytoprotective, etc. (Table S1 in Supplementary Materials) have been widely reported [4,6,7,8,9,10,11,12,13,14,15,16,17,18,19,20,21,22,23,24,25,26,27,28].

As the extracts were prepared under different conditions, the results, e.g., total phenolic content (TPC) or concentrations of individual polyphenolic compounds significantly differed. On the other hand, when several raspberry cultivars were analyzed according to the same protocol, both TPC and composition of leaf extract components varied depending on the cultivar, stage of development, and environmental conditions [6,11,29]. The major characteristic compounds found in leaf extracts were derivatives of ellagic acid, phenolic acids, quercetin and its derivatives, quercitrin, kaempherol, and various catechins [20,21,22,29,30,31]. Dominating compounds identified in young shoots were found to be ellagitannin sanguiin H-6 and free ellagic acid followed by catechins, quercetin derivatives, and phenolic acids [4,32]. Raspberry bud extracts also have gained attention as a source of bioactive compounds such as catechins, flavonols, and phenolic acids [33]. Due to different protocols of experiments, it was not possible to establish the correlation among principal characteristic compounds in leaf extracts and those in shoot or bud extracts.

The health benefits of plant extracts, including antioxidant activity, are thought to be related to a variety of polyphenolic compounds [34,35,36,37,38,39,40,41]. An antioxidant (a definition used mostly in biological context) is defined as an easily oxidizable substance that, when present in low concentrations compared to those of other oxidizable constituents, protects the latter compounds from oxidation. Evaluated in vitro mostly by spectrophotometric assays, the abilities of polyphenols to scavenge free radicals 1,1-diphenyl-2-dipicrylhydrazyl (DPPH^●^), 2,2-azino-bis(3-ethylbenzothiazoline-6-sulfonic acid (ABTS^●+^), or reactive oxygen species (ROS) or to form complexes with transition metals have been considered as their potential antioxidant capacities [40,42].

Electrochemical techniques such as cyclic voltammetry, differential pulse, or square wave voltammetry have also been employed for evaluation of antioxidant properties of beverages, plant extracts, or individual polyphenols [43,44,45,46,47,48,49,50,51,52,53,54,55,56]. Electrochemical approaches are based on the physical-chemical properties of the compounds and can therefore be considered as a direct test for antioxidant properties. The results derived from electrochemical measurements correlated with the TPCs and radical scavenging activities in herb extracts [45], dark fruit juices [49], herbal tea infusions [50], Croatian red wines [51], and Sauvignon blanc grape juice [53], although some discrepancy between electrochemical data and phenolic content in white, tawny, or muscatel wines was also reported [54].

In addition to polyphenols as antioxidants, various polyphenols are known to exhibit prooxidant activity leading to formation of ROS such hydrogen peroxide, hydroxyl radical (OH^●^), or superoxide anion (O^2●−^) [57,58,59,60,61]. There have also been reports on dual—antioxidant and prooxidant—activities of common medicinal herbs [62] or popular catechin-rich beverages (green or black tea, coffee) [57,63,64] related to scavenging and production of hydrogen peroxide.

Raspberry berries, leaves, and stems are used in folk medicine in Lithuania for self-treatment of the common cold and flu [65]. Some results of the investigation of TPC and antioxidant properties of extracts of raspberry leaves collected from various localities in Lithuania at different harvesting times have been already presented [11,29]. To the best of our knowledge, studies on red raspberry stem extracts have not yet been reported. For healing purposes, raspberry stems are harvested in the cold season when there are no leaves, usually just before use. This work was designed to investigate the properties (TPC, antioxidant, and prooxidant activities) of stem extracts employing spectrophotometric and electrochemical assays. The stems were collected at different time of the year in order to find out whether the collection time was critical for the properties of the extracts.

## 2. Results

Preliminary study on *R. idaeus* leaf and stem extracts revealed that methanolic extracts and decoctions contained similar amounts of residuals (Table S2).

### 2.1. TPC (Total Phenolic Content)

TPCs in raspberry stem, leaf and bark extracts determined by Folin-Ciocalteu method [66] are presented in Table 1.

The concentrations of polyphenolics in the extracts of dried raspberry stems varied with stem harvesting time, the mode of extract preparation, and the freshness of the sample. Leaf and stem samples should be analyzed within 2 months after gathering since prolonged storage of the plant material resulted in significant decrease of concentrations of polyphenolics (Figure S1 in Supplementary Materials).

After 2 years of storage, up to 35% of polyphenolic compounds remained in winter stems (Table 1). The quantities of polyphenolic compounds (Table 1) in extracts increased with higher extraction temperatures and assistance of ultrasound. Extraction at 50–55 °C, 70–75 °C, or by boiling resulted in 2 to 3-fold increase of TPC compared to that obtained at room temperature. Extractions of peeled bark prepared with the assistance of ultrasound at room temperature contained considerably larger quantities of polyphenols (597.37 ± 8.79 mg/L of gallic acid equivalent) even without heating.

Bark extract for electrophoresis and two fractions (protein FA and polyphenolic FB) separated from it were needed in order to further elucidate the role of protein in H_2_O_2_ scavenging. The protein fraction FA was not completely purified from phenolics whereas FB fraction did not contain proteins (Figure S2 in Supplementary Materials).

### 2.2. Chemical Composition of R. idaeus Methanolic Extracts

Ten compounds were identified tentatively in the raspberry leaf and stem methanolic extracts. All constituents were detected by TOF in positive ionization mode and by DAD (Table 2). Some compounds provided *m*/*z* ions by both (positive and negative) ionizations.

### 2.3. Antioxidant Activity

#### 2.3.1. DPPH Radical-Scavenging Activity

All investigated raspberry leaf, stem, and bark extracts showed DPPH radical-scavenging activity, ranging from 0.13 ± 0.01 to 1.90 ± 0.04 mmol/L (TROLOX equivalent) (Table 3).

Leaf and bark extracts containing the highest quantity of polyphenolic compounds (Table 1) showed the highest radical-scavenging activity. The activity of extracts prepared from winter stems was significantly lower compared to that of spring stem and leaf extracts. DPPH^●^ scavenging activity of extract of stored stems was approximately 0.75-fold lower (Table 1) compared to that of extract of “freshly collected” winter stems.

Investigating the relationship between TPCs found in raspberry extracts and their free radical scavenging activity found a moderate positive correlation r = 0.44 (when n = 10, Pearson correlation coefficient), indicating an average correlation between these two variables. These data suggest that antioxidant activity of above-mentioned extracts cannot be unambiguously attributed only to polyphenol content but also can be related to other direct or indirect factors.

#### 2.3.2. Electrochemical Assays

##### Cyclic Voltammetry

Cyclic voltammetry is the most widely used technique to obtain the data about redox active substances in the solution [67]. The working metal or carbon-based electrode is immersed in the sample and its potential is scanned to the positive direction. As soon as the potential of the electrode reaches the oxidation potential of a sample constituent, the oxidation of the compound occurs: the lower the potential of oxidation, the more powerful reducing, i.e., antioxidant, properties of the compound. The oxidation (anodic) peak potential value (E_pa_) depends on chemical structure of the electroactive substance, electrode material, pH value, and composition of the solution. The magnitude of the oxidation (anodic) peak current (I_pa_) at E_pa_ is related to the concentration of the electroactive compound. During the reverse scan, reduction currents are registered. The presence of reduction (cathodic) peaks I_pc_ at reduction (cathodic) potentials E_pc_ in the reverse scan shows the reversibility of the redox reaction, i.e., oxidized compounds generated in the forward scan are reduced in the reverse scan [62]. The E_pa_ value was suggested as a criterion for antioxidant activity: compounds with oxidation potential values E_pa_ < 0.45 V were considered as antioxidants [46].

**Figure 1:**
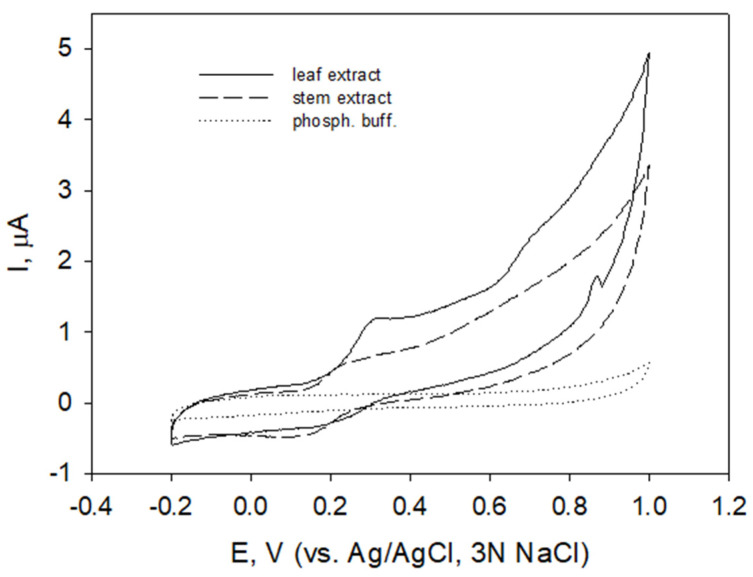
Cyclic voltammograms of carbon paste electrodes in *R. idaeus* leaf and stem extracts in phosphate buffer pH 6.0 (as indicated), potential scan rate 50 mV/s.

Cyclic voltammograms of carbon paste electrode in leaf and stem extracts of raspberry (shown for plant material collected in August) is presented in Figure 1. Both oxidation and reduction currents were registered at relatively low potentials falling in region 0 to 0.4 V and indicating the presence of possibly the same electroactive species with reversible redox process. Voltammetric profiles of extracts prepared from stems collected in spring and winter (not shown) were similar but differed in peak current values.

##### Differential Pulse Voltammetry

**Figure 2:**
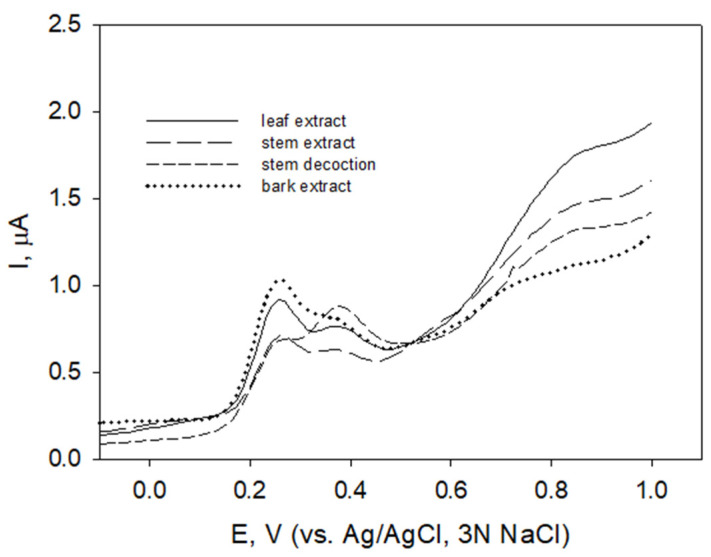
Differential pulse voltammograms of carbon paste electrode in *R. idaeus* leaf, stem, and bark extracts (diluted 1:5) prepared at room temperature and stem decoction in phosphate buffer pH 6.0; potential step 4 mV, pulse width 50 ms, pulse period 200 ms, pulse amplitude 50 mV.

Differential pulse voltammetry allows obtaining improved resolution between the species with comparable redox potentials [67]. Differential pulse voltammograms of raspberry leaf, stem (both “freshly collected” and stored for 2 years), bark extracts, and stem decoction revealed similar voltammetric profiles in the potential region 0.1 to 0.5 V (Figure 2, shown for stems and leaves collected in August, and bark peeled from stems collected in December) with peak currents at E_pa1_ 0.25 V and E_pa2_ 0.38 V. The voltammograms differed in peak current values at these potentials.

The increase of the currents at potential region above 0.6 V indicated the presence of compounds with relatively high oxidation potentials.

**Figure 3:**
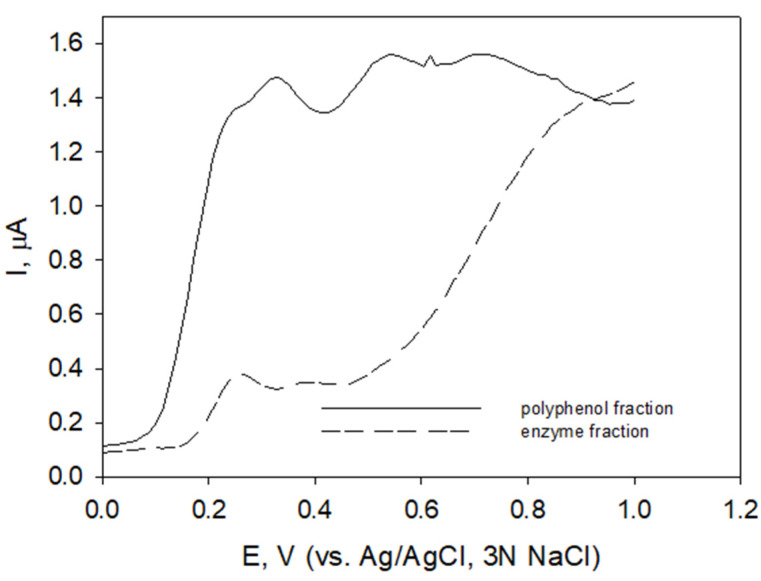
Differential pulse voltammograms of carbon paste electrode in polyphenolic fraction FB (solid line) and enzyme fraction FA (dashed line) of *R. idaeus* bark extract, phosphate buffer pH 6.0; potential step 4 mV, pulse width 50 ms, pulse period 200 ms, pulse amplitude 50 mV.

Differential pulse voltammograms of protein fraction FA and polyphenolic fraction FB obtained from bark extract are shown in Figure 3.

The voltammetric profile of polyphenolic fraction changed after protein elimination procedure, especially in the potential region E > 0.5 V, (Figure 3, solid line) compared to that obtained for enzyme fraction (Figure 3, short-dashed line).

##### H_2_O_2_ Scavenging Activity

**Figure 4:**
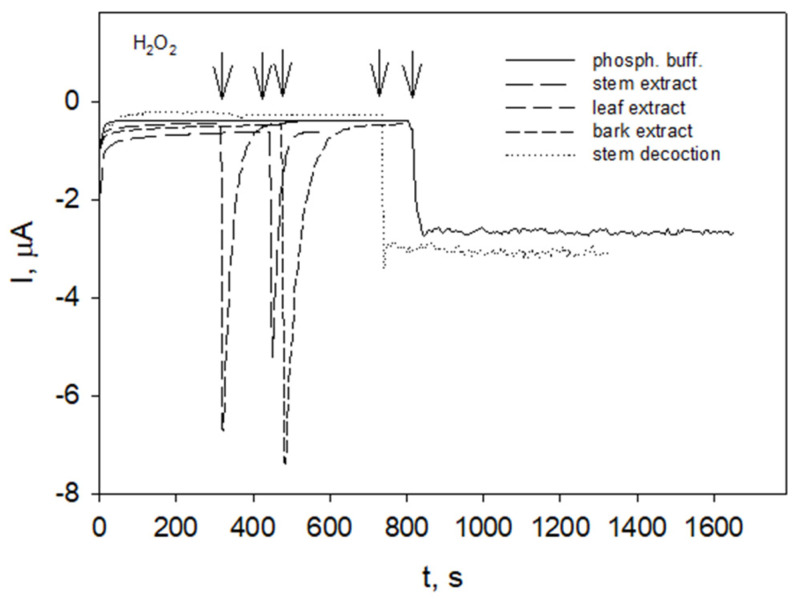
Responses of GC/PB in phosphate buffer pH 6 and various *R. idaeus* extracts (as indicated) obtained at room temperature to the additions of H_2_O_2_, operating potential 0.0 V. Arrows indicate the moments of H_2_O_2_ addition. Plant material (leaves and stems) collected in August and bark peeled from stems collected in December.

Electrocatalytical properties of Prussian Blue (PB) allow the reduction of hydrogen peroxide at potentials around 0.0 V, thus excluding the influence of other electroactive species [68]. The Prussian Blue-modified electrode (GC/PB) was held at a constant potential of 0.0 V until a steady state of the background current was achieved. After injection of hydrogen peroxide into the phosphate buffer a steady cathodic current related to hydrogen peroxide reduction at GC/PB was registered (Figure 4, solid line). When hydrogen peroxide was injected into raspberry stem, leaf, and bark extracts (Figure 4, long-dashed, medium-dashed and short-dashed line, respectively), the hydrogen peroxide-induced reduction currents immediately started to decay and within 3 min. reached the initial steady state values. Disappearance of reduction current indicated that there was no electroactive substance, i.e., hydrogen peroxide was scavenged by the extracts.

Similar disappearance of hydrogen peroxide reduction current (not shown) was observed for extracts of raspberry herbal material collected in May unless the extracts were exposed to temperatures above 70 °C.

In order to elucidate whether the loss of peroxide-scavenging property was related to the presence temperature-sensitive enzymes, protein (enzyme) fraction from raspberry bark extract was isolated by means of passing the extract through membrane filters and subsequent concentration/dilution procedures. Gel electrophoresis showed that the sample containing proteins (Figure S2 in Supplementary Materials, band FA) and the solution bypassed centrifugal filter (Figure S2 in Supplementary Materials, band FB) were markedly different. The proteins were eliminated from the latter sample. Both samples contained polyphenols (Table 1).

**Figure 5:**
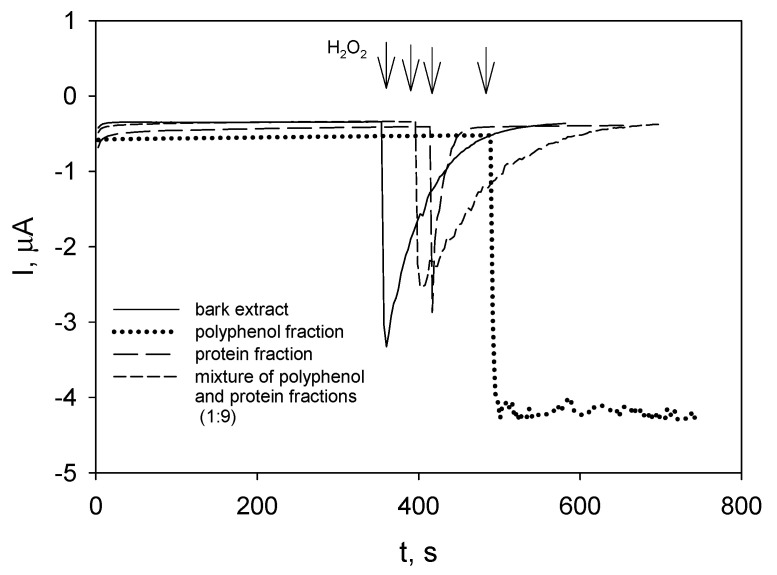
Responses of the GC/PB to the additions of H_2_O_2_ to initial *R. idaeus* bark extract, polyphenolic, and enzyme fractions obtained from bark extract and their mixture (as indicated); phosphate buffer pH 6.0; operating potential 0.0 V.

As indicated by decaying currents, initial raspberry bark extract (Figure 5, solid line) and enzyme (protein) fraction (Figure 5, long-dashed line) scavenged hydrogen peroxide, whereas the steady state reduction currents were recorded in polyphenolic fraction (Figure 5, dotted line). After mixing polyphenolic fraction with protein (enzyme) fraction, the decay of reduction current was obtained again (Figure 5, short-dashed line). The latter mixture contained relatively low concentration of polyphenols compared to that of bark extract; nevertheless, the time course of the current decay was similar.

**Figure 6:**
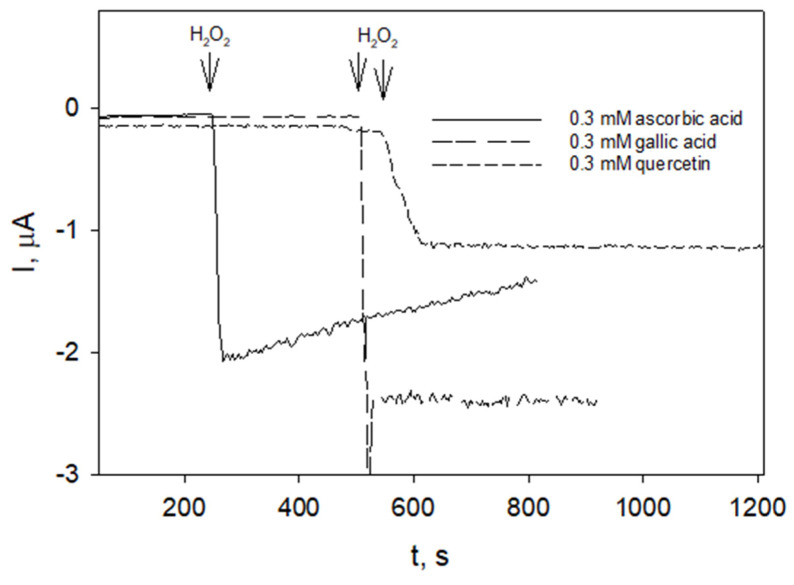
Responses of the GC/PB to the additions of H_2_O_2_ into solutions of ascorbic acid, gallic acid, and quercetin (as indicated) in phosphate buffer pH 6.0, operating potential 0.0 V.

The solutions of well-known antioxidants such as ascorbic acid, gallic acid, and quercetin were taken for comparison of H_2_O_2_ scavenging activity (Figure 6). Only ascorbic acid showed some activity in scavenging H_2_O_2_ (Figure 6, solid line).

### 2.4. Prooxidant Activity

Prooxidant activity of raspberry stem (collected in December) extracts and infusions related to production of hydrogen peroxide was tested at pH 4.6 and pH 7.3. The amount of hydrogen peroxide produced in 30 min is presented in Table 4.

The amounts of hydrogen peroxide formed in raspberry extracts and infusions in phosphate buffer at pH 7.3 were, respectively, 1.5- and 4.9-fold higher compared to those obtained at pH 4.6.

## 3. Discussion

Preliminary study on *R. idaeus* leaf and stem extracts was performed in order to determine dry content and to choose the most effective solvent for extraction. The amounts of the medium-polarity constituents were approximately the same in both the stems and leaves of *R. idaeus* (Table S2). The content of polar molecules in the stems was low compared to the leaves. The total content of compounds of different polarity in raspberry stem extracts was as follows: medium polarity compounds > polar components > non-polar compounds. The total content of components of different polarity in the raspberry leaf extracts was in the following order: polar compounds > components of medium polarity > non-polar compounds. *R. idaeus* leaf and stem methanolic extracts and decoctions contained similar amounts of residuals (Table S2); therefore, these solvents were chosen for further experiments.

Previously reported TPC or concentrations of individual polyphenolic compounds in raspberry leaf extracts significantly differed (Table S3 in Supplementary Materials, shown for TPC) since the extracts were prepared under different conditions using various solvents (water, methanol, ethanol, or mixtures of alcohols with water at different ratios), different extraction times, and temperatures.

Antioxidant properties of plant extracts are related to TPC that is dependent on solvents (aqueous or organic) used for extraction [5,14,69,70,71,72]. In this research, phosphate buffer was used as a solvent in order to be further able to perform electrochemical experiments with the same extracts.

In order to find out how the quantities of polyphenolic compounds influence the antioxidant and prooxidant properties of raspberry stem extracts, different extraction conditions were applied. As was expected, the TPC (expressed in gallic acid equivalent) increased with assistance of ultrasound and higher extraction temperatures (Table 1, shown for stems harvested in August). Comparing the results obtained, for example, at room temperature, the extracts of stems collected in winter contained approximately 1.7 times more polyphenolics than those found the in the stems gathered in summer. Leaf extract contained relatively high amounts of polyphenolic compounds even without heating or ultrasound assistance. Another expectation that the bark extract would contain more polyphenolics than the stem extract was also met (Table 1). TPC in the bark (peeled from the winter stems) extract was approximately 2.1-fold higher compared to that in the stem extract obtained under the same conditions. Prolonged storage (approximately 2 years at room temperature in the darkness) of stems revealed the loss of polyphenolics (Table 1).

Correlation between radical scavenging activity and TPC was better for extracts prepared under different conditions from the same plant material (e.g., bark extract and its fractions) compared to that when extracts were prepared from plants harvested at different time of the year (Table 1 and Table 2). Scavenging activity of bark extract appeared to be significantly (approximately 9-fold) higher compared to that of extract of winter stems obtained under the same conditions while the concentrations of polyphenols in these two extracts differed only 2-fold (Table 2). However, although extracts prepared from stems stored for 2 years contained approximately one third of polyphenolic compounds compared to extracts of “freshly collected” stem, DPPH^●^ scavenging activity of extracts of stored stems was only approximately 0.75-fold lower (Table 1 and Table 2).

Extracts prepared from stems and leaves collected in spring showed significantly higher radical-scavenging activity compared to that of winter stem extracts. TPC in leaf extract was approximately 5.4-fold higher than that in stem extract (Table 1) prepared under the same conditions, while scavenging activity was only 1.4-fold higher. Outcomes from radical scavenging activity and composition of raspberry leaves from different locations in Lithuania [11] revealed that all ethanolic extracts prepared from raspberry leaves were DPPH radical-scavenging active and overall correlated both with TPC and another antioxidant (ABTS) test. However, no obvious dependence of scavenging activity and amount polyphenolics on plant harvesting time was reported.

The methanolic extracts of young, non-lignified shoots of raspberry were found to be effective DPPH radical scavengers [4]. However, due to different protocols of assays, activities of shoot and extracts of raspberry stems gathered in spring and used in this research cannot be compared.

The antioxidant activity of extracts prepared from *R. idaeus* varied depending both on the part of the plant used and on the conditions of extract preparation (Table 3). The bark extract (2018 December) prepared by ultra-sonication showed almost nine times higher radical-scavenging ability than the stem extract (2018 December) produced by the same method. The latter extract exhibited an antioxidant activity up to two times lower than the stem extract prepared by infusion. Comparing the antioxidant activity of all the raspberry extracts tested, as determined by the DPPH^●^ assay, it is evident that the bark extracts (≤1.47 ± 0.24 mmol/L TROLOX equivalent) are richer in compounds capable of scavenging free radicals. Similar activity was observed for the extracts prepared at room temperature from stems (2019 May) and leaves (2019 May) (1.18 ± 0.07 and 1.63 ± 0.10 mmol/L TROLOX equivalent, respectively). Data on antioxidant activity of various *R. idaeus* extracts suggest that bark extracts are the most effective DPPH radical scavengers.

Spectrophotometric radical-scavenging DPPH^●^ assay in vitro is a very popular antioxidant test and actually often correlates with TPC. However, critical evaluation of the assay pointed to its main limitation, i.e., DPPH is not found in living organisms. Furthermore, the following other considerations argue against direct polyphenol reactions with radicals in vivo: the low concentrations of polyphenols in tissues, high level of metabolism and biotransformation that polyphenols undergo in the organism, and slow action (minutes or hours) as a radical scavenger of an antioxidant must be irrelevant in vivo in cells or even in situ in foods, etc. [73].

As an alternative to radical-scavenging assay, electrochemical approaches based on the chemical-physical properties of the compounds were applied to test antioxidant properties. In the region of potentials of interest (E_pa_ < 0.45 V) related to antioxidant properties of compounds [46], similar voltammetric profiles (Figure 1 and Figure 2, solid and long-dashed lines, shown for extracts of stems and leaves) indicated that the extracts contained the same polyphenolic compounds characterized by E_pa1_ 0.25 V and E_pa2_ 0.38 V. Different ratios of I_pa_ values at these potentials in stem decoction (Figure 2, short-dashed line) may indicate different ratios of extracted certain polyphenols. To obtain a comparable voltammogram, raspberry bark extract was diluted (1:5) prior to measurements. Polyphenols with pH-dependent oxidation potentials falling in the potential region 0.2 to 0.4 V at pH 6 are possibly compounds containing a flavonoid structure with catechol or galloyl moieties (catechins, epicatechin, quercetin) [43,44,73]. Catechins show a reversible behavior at carbon-based electrodes [74]. Direct comparison of obtained E_pa_ values with literature data is rather complicated since the conditions of the experiment (electrode material, ionic strength of the solution, the presence of organic solvent, concentration of electroactive substance, etc.) may cause peak shifts [75].

Voltammetric profiles of polyphenol and enzyme fractions obtained from raspberry bark extract (Figure 3) differed. Higher I_pa_ values of polyphenolic fraction correlated with TPC in these fractions (Table 1).

ROS (reactive oxygen species) are inevitably produced as a by-product of normal aerobic metabolism and could be injurious for cells when present in excess under stress conditions [76]. It was considered reasonable to evaluate antioxidant properties of polyphenols and plant extracts by their capabilities to scavenge those ROS [77]. To assess the H_2_O_2_-scavenging activity of polyphenols and/or extracts of plants, several methods have been developed including colorimetric enzyme peroxidase-based detection systems employing the oxidation of substrates by H_2_O_2_ [78,79,80,81] and non-enzymatic methods such as direct spectrophotometric determination at a wavelength of 230 nm [82,83], chemiluminometric detection of background emission decrease when an antioxidant in the sample eliminated H_2_O_2_ [84], amperometric assay by monitoring of oxygen evolution at an oxygen electrode system [85], polarographic assay of H_2_O_2_ at dropping Hg electrode based on the formation of the mixed mercury complex and its decrease upon addition of polyphenols [86], and kinetic approach by monitoring kinetics of hydrogen peroxide scavenging at Prussian Blue (PB)-modified electrodes [87].

Electrocatalytical properties of PB allow the reduction of hydrogen peroxide at potentials around 0.0 V, thus excluding the influence of other electroactive species [68]. Although *R. idaeus* leaf and bark extracts contained higher concentration of polyphenols compared to that in stem extract (Table 1), the polyphenol concentration was not essential for hydrogen peroxide scavenging capability of extracts (Figure 4, long-, medium-, short-dashed lines). The extracts prepared from stored winter stems, although containing relatively low concentrations of polyphenols, appeared to possess peroxide scavenging activity (not shown). In the case of raspberry stem decoction (Figure 4, dotted line) or infusion (not shown) of stem extract obtained at 70 °C (not shown), the reduction currents remained stable, indicating the presence of hydrogen peroxide in the solution, i.e., the hydrogen peroxide-scavenging ability of the extract was lost. This suggested that the extracts possibly contain the enzymes (such as peroxidases) that contribute to elimination of hydrogen peroxide and lose this ability at elevated temperatures.

Experiments with polyphenol and protein-containing fractions of bark extracts showed that the presence of certain protein/proteins was essential for hydrogen peroxide scavenging ability of extracts (Figure 5). To find out what specific protein was responsible for hydrogen peroxide scavenging was not the purpose of this investigation, especially since the extracts of other plants also possessed peroxide-eliminating activity (the research is in progress).

The reports on hydrogen peroxide-scavenging activities of catechins, quercetin, or gallic acid usually present in raspberry leaf extracts are contradictory. When measured by using peroxidase-based assay, catechin, epicatechin, quercetin [84], or gallic acid [80] were found to be effective scavengers of hydrogen peroxide. Catechins isolated from green tea were found to scavenge hydrogen peroxide directly in a concentration-dependent manner [88]. In contrast, amperometric measurements using oxygen electrode system revealed that gallic acid and catechins did not react with hydrogen peroxide [85]. The nonenzymatic oxidation of quercetin by hydrogen peroxide was negligible [78]. Fast oxidation of quercetin could be observed in the presence of horseradish peroxidase or soluble fraction of *Shefflera arboricola* leaf extract containing peroxidase [78]. The measurements employing GC/PB electrodes revealed that hydrogen peroxide was not scavenged by gallic acid or quercetin as indicated by steady states of reduction currents (Figure 6, long- and short-dashed lines, respectively). However, hydrogen peroxide reduction current decayed in the presence of ascorbic acid (Figure 6, solid line) which is known to react with hydrogen peroxide directly [89,90]. The time taken for the peroxide reduction current to decrease to the initial value was significantly shorter, by approximately 2 to 3 min (Figure 4, long-, medium-, short-dashed lines), than that taken for this current to decrease using ascorbic acid (Figure 6, solid line). An approach to evaluate antioxidant activity by monitoring kinetics of hydrogen peroxide scavenging was formerly reported for various food samples (juices, several wine samples, juice sublimates) [87]. Some food products did not show the ability to remove peroxide. The conditions of food sample manufacturing (whether they were subjected to elevated temperatures or not) or compositions (whether they contained, e.g., ascorbic acid) are unknown, making it difficult to compare with the results obtained with raspberry extracts in this study.

Contrary to unfavorable effect of elevated temperatures on hydrogen peroxide scavenging activity, the amount of generated hydrogen peroxide (prooxidant activity) was higher in *R. idaeus* stem infusion compared to that in stem extract (Table 4) and correlated with TPC (Table 1). Solution pH was another important factor affecting the generation of hydrogen peroxide: the concentrations of hydrogen peroxide formed in extract and infusion at pH 7.3 in 30 min. were, respectively, 1.5- and 4.9-fold higher compared to those obtained at pH 4.6. The increase of peroxide quantity at more alkaline pH value is in agreement with formerly reported higher prooxidant activity of aqueous extracts of *Rosa canina* L., *Hypericum perforatum* L., *Rhodiola rosea* L., *Gentiana lutea* L. at more alkaline pH values [62]. Catechins efficiently generate hydrogen peroxide under alkaline conditions [57]. Raspberry leaves and young shoots are known to contain catechins [4,14,32] as well as the extracts of *R. canina* [91] or *R. rosea* [92]. Catechins are among substances determined in methanolic extracts of raspberry stems/leaves, and the results of cyclic voltammetry indicate the possible presence of catechins in the extracts, making it a fairly probable cause of hydrogen peroxide formation responsible for the prooxidant activity of raspberry stem extracts.

## 4. Materials and Methods

### 4.1. Plant Material

Two-year-old stems with leaves of raspberry plants growing wild near Vilnius city (Lithuania, 54°42′47.4″ N 25°22′13.0″ E) were collected after harvesting the berries in late August and December (2018), and just before flowering in early May (2019). The area of the investigated population was up to 100 m^2^. Raw material was taken immediately to the laboratory and dried at room temperature (20–25 °C) under shade conditions for 3 to 4 weeks. For some experiments, bark peeled from the stems was used.

Plant material has been identified by dr. M. Rasimavičius, and voucher specimen was deposited at the Vilnius University Herbarium (WI, Lithuania) with a code number P33611.

### 4.2. Preparation of Extracts

#### 4.2.1. Preparation of Extracts with Different Solvents for Determination of Dry Content

Lixiviation procedure was applied for preparing various *R. idaeus* extracts according to Pharmacopeia. Pulverized herbal material (20 g) was placed in a glass column (diameter approximately 3–4 cm), 200 mL of dichloromethane were added and leaved for maceration at room temperature. After 24 h, dichloromethane was poured and 200 mL of filtrate was collected, then solvent was removed by rotary evaporation. Residues, containing mainly non-polar constituents from the plant material, were weighted. In order to collect moderate polar and polar compounds, the procedure was repeated on the same herbal material with methanol and mixture of methanol and water (1:1). Additionally, decoction procedure was applied. 20 g of *R. idaeus* leaves and 300 mL of distilled water were boiled for 15 and 30 min. Stems (20 g) were boiled for 30 min. Solutions were decanted and filtrated on cotton. Filtrates were lyophilized under temperature conditions from −40 °C to 30 °C.

#### 4.2.2. Preparation of Extracts for Electrochemical Measurements

Stems and leaves were separated before extract preparation and ground in a mill. Then, 5 g of stem, leaf, or bark powder were placed in 75 mL of phosphate buffer at pH 6.0 consisting of 0.05 mM KH_2_PO_4_ and 0.1 M KCl (both from Fluka). The pH value was adjusted with KOH (Fluka). Extractions were performed at room or higher temperatures with or without ultrasound for 30 min. Decoctions were prepared by boiling 5 g of ground plant material in 75 mL of phosphate buffer pH 6.0 for 30 min. Infusions were made from 75 mL of boiling phosphate buffer and 5 g of plant material, and thereafter allowed to cool to room temperature.

#### 4.2.3. Preparation of Extracts for HPLC-DAD-TOF Analysis

Samples of air-dried leaves and stems of *R. idaeus* were ground into a homogenous powder and protected from light and humidity until analysis. Preparation of extract was made according to the Pharmacopeia requirements. For extraction, 1 g of crushed herbal material and 10 mL of solvent (mixture of water and methanol (1:1)) were used. Extraction procedure was performed in an ultrasonic bath at room temperature for 15 and 30 min, respectively, for raspberry leaves and stems. Mixture was filtrated through a filter paper for qualitative analysis (pore size 11 µm).

### 4.3. Determination of TPC

TPC in *R. idaeus* extracts was determined using Folin-Ciocalteu assay [66].

First, 20 μL of raspberry leaf, stem, or bark extract and 1580 μL of distilled water was added to 100 μL Folin-Ciocalteu reagent and 300 μL of Na_2_CO_3_ (20% *w*/*v*). The mixture was left in the darkness at room temperature for 2 h. The absorbance at 765 nm wavelength was measured using the spectrophotometer (UV/Vis Lambda 25, Perkin Elmer, Buckinghamshire, UK). The results are expressed as mg/L gallic acid equivalent. Calibration curve used for calculations (Figure S3 in Supplementary Materials) was obtained using different concentrations of gallic acid 0.00; 50; 100; 150; 250; and 500 mg/L. All measurements were done in triplicate.

### 4.4. HPLC-DAD-MS (TOF) Analysis of R. idaeus Extracts

Different extracts of *Rh. idaeus* leaves and stems were analyzed by HPLC technique, using a system HPLC/Diode Array Detector (DAD)/Time of Flight (TOF) (Agilent 1260 Infinity (Agilent Technologies, Waldbronn, Germany) and Agilent 6224 TOF (Agilent Technologies, Santa Clara, CA, USA) equipped with a reverse phase column ZORBAX Eclipse XDB (C18, 5 μm particle size, 150 × 4.6 mm, Agilent Technologies, Santa Clara, CA, USA). The column temperature was maintained at 35 °C. Gradient system was applied: A (deionized water, containing 0.1% of formic acid) and B (acetonitrile, containing 0.1% of formic acid). Chromatographic separation was performed at a flow rate of 0.7 mL/min in the HPLC system by the following stepwise gradient elution method: initial 40% (A)/60% (B); from 0 to 2 min and 40% (A)/60% (B); from 2 to 9 min: 40% (A)/60% (B) to 30% (A)/70% (B); from 9 to 13 min isocratic mode at 30% (A)/70% (B); from 13 to 29 min: 30% (A)/70% (B) to 10% (A)/90% (B); from 29 to 35 min: 10% (A)/90% (B) to 40% (A)/60% (B). Ionization was performed by electrospray ionization interface (ESI) in positive or negative mode. Sample volume of 10 and 15 µL for raspberry leaf and stem extracts, respectively, was injected by auto-sampler.

MS (TOF) acquisition parameters were as follows: mass range 100–1700 *m*/*z*, rate 1.42 spectra/s, time 704.2 ms/spectrum. Ionization source conditions were: drying gas temperature 300 °C, drying gas flow rate 3 L/min, nebulizer 15 psig, fragmentor voltage 125 V, skimmer 65 V. To assure the mass accuracy of recorded data, continuous internal calibration with reference masses *m*/*z*: 121.050873, 149.02332, 322.048121, 922.009798, 1221.990637 and 1521.971475 (as per instrument standards, ref. nebulizer 5 psig) was performed.

### 4.5. Antioxidant Activity Tests

#### 4.5.1. Spectrophotometric DPPH Radical Scavenging Assay

First, 6 × 10^−5^ M stock solution of DPPH^●^ was obtained by dissolving 2,2-diphenyl-1-picrylhydrazyl in methanol. The working solution was prepared by diluting stock solution with methanol to obtain an absorbance value of 0.730 ± 0.02 at 515 nm. Raspberry stem and leaf extracts for analysis were diluted 1:50 with a mixture of methanol and water (80:20); 0.1 mL of prepared sample was allowed to react with 3.9 mL of working DPPH^●^ solution in the darkness for 30 min. Thereafter, the absorbance of reacted mixture was measured. The results are expressed in mmol/L TROLOX equivalent. Then, 5 mg of TROLOX (±)-6-hydroxy-2,5,7,8-tetra-methylchromane-2-carboxylic acid) was dissolved in methanol and water solution (70:30) and diluted to 100 mL. Five different concentrations from this solution were prepared (200, 100, 50, 25, and 12.5 mmol/L), and 0.1 mL of each TROLOX solution was allowed to react with 3.9 mL of working solution of DPPH^●^. The absorbance values were measured at 515 nm after 30 min. The absorbance was measured using the spectrophotometer (UV/Vis Lambda 25, Perkin Elmer, Buckinghamshire, UK). Linear calibration curves (Figure S4 in Supplementary Materials) were obtained, and their parameters were used for further calculations of antioxidant capacity. All measurements were completed in triplicate.

#### 4.5.2. Electrochemical Tests

Amperometric measurements were performed using BAS-Epsilon Bioanalytical system (West Lafayette, IN, USA). A conventional three electrode cell contained carbon paste or Prussian Blue-modified glassy carbon electrode (GC/PB) as working electrodes, platinum as an auxiliary electrode and Ag/AgCl, 3 M NaCl as a reference electrode.

##### Cyclic and Differential Pulse Voltammetry

Carbon paste electrode was prepared by thoroughly mixing 200 mg of graphite powder with 100 µL of paraffin oil (both from Fluka). The paste was packed into the cavity of a homemade electrode consisting of a plastic tube (2.9 mm) and a copper wire served as an electrode contact. The surface of the electrode was thereafter smoothened on a white paper. Cyclic voltammograms were recorded in the potential region −0.2 to 1.0 V at potential scan rate 50 mV/s. Differential pulse voltammograms were recorded at potential step 4 mV, pulse width 50 ms, pulse period 200 ms, pulse amplitude 50 mV.

##### Hydrogen Peroxide Scavenging Test

Prior to electrodeposition of PB, the glassy carbon electrode was polished with Al_2_O_3_ to mirror finish and sonicated in water for 2 min. PB was electrodeposited from a solution containing 2.5 mM FeCl_3_, 2.5 mM K_3_[Fe(CN)_6_], 0.1 M KCl (all from Fluka), and 0.1 M HCl (Reakhim, Russia, Moscow) by applying 400 mV for 40 s. Thereafter the electrode was transferred to solution containing 0.1M KCl and 0.1 M HCl and cycled between 350 mV and −25 mV 25 times (potential scan rate 25 mV/s).

To assess hydrogen peroxide scavenging activity, GC/PB was held in phosphate buffer pH 6.0 or raspberry leaf, stem, and bark extracts or 0.3 mM solutions of quercetin, gallic acid, or ascorbic acid at 0.0 V until a steady state of the background current was achieved. Hydrogen peroxide solution was then added to a final concentration of 0.15 mM.

### 4.6. Prooxidant Activity Test

First, 100 µL of raspberry stem extracts and infusions prepared in phosphate buffer pH 4.6 and 7.3 were diluted with appropriate buffers to a final volume of 2 mL and incubated at room temperature for 30 min. After incubation, the samples were 100-fold diluted and 200 μL of the samples were mixed with 1.8 mL of FOX reagent (250 μM FeSO_4_, 25 mM H_2_SO_4_, 100 μM xylenol orange and 100 mM sorbitol). The reaction mixture was thereafter incubated at room temperature for 30 min. The absorbance of the solution was measured at 580 nm wavelength. For calibration curve (Figure S5 in Supplementary Materials), 1 to 100 μM hydrogen peroxide solutions were used.

### 4.7. Isolation of Proteins from Raspberry Bark Extract

Dry raspberry bark was ground to powder and mixed with phosphate buffer pH 6.0 at a ratio of 1 g of plant material to 15 mL of buffer. The crude extract was kept at room temperature for 60 min. Thereafter the mixture was filtered by passing solution through membrane filters (pore sizes 5 µm). Liquid fraction was replaced in the centrifugal filter (Amicon ^®^ Ultra-15, Merck-Millipore) and centrifuged at 3220× *g* until the volume of protein solution decreased 9 to 10-fold. Concentrate was diluted up to a primary volume with buffer. Concentration/dilution procedure was repeated two times. The sample containing proteins and the centrifuged solution were taken for polyacrylamide gel electrophoresis.

### 4.8. Polyacrylamide Gel Electrophoresis

Sodium dodecyl sulfate polyacrylamide gel electrophoresis (SDS-PAGE, Bio Rad PowerPac Basic, Bio-Rad Laboratories, Hercules (HQ), CA, USA) was used to obtain high resolution analytical separation of mixtures of proteins. Denaturing SDS-PAGE was performed according to the Thermo Fisher Scientific^®^ specifications. In brief, 10 μL of protein sample were mixed with 5 μL of Laemmli buffer (solution contained 6% SDS, 30% glycerol, 300 mM DTT, 0.06% bromphenol blue and 240 mM Tris HCl, pH approx. 6.8) and heated at 95 °C for 10 min. Samples were loaded into freshly prepared 12% Tris 1.0 mm gel. Then, 2 μL of PageRuler^TM^ Prestained Protein Ladder (Thermo Fisher Scientific Baltic, Vilnius, Lithuania) were loaded in each gel run. Electrophoresis was performed at room temperature for approximately 60 min using a constant voltage (180 V) in 1X solution of Tris-Glycine-SDS running buffer until the dye front reached the end of the 60 mm gel.

An appropriate mass of Bovine Serum Albumin (BSA) protein was loaded in each lane of SDS-PAGE. The amount of extracted protein could be evaluated by visual comparison of band of unknown protein versus BSA band.

### 4.9. Statistical Analysis

The obtained results were statistically processed by calculating the Pearson correlation coefficient (*r*); the results were expressed as mean values, range intervals, and standard deviation (SD) values, using XLSTAT (trial version, Addinsoft 2014, Paris, France). Evaluation of significant differences between extracts for each analysis of antioxidant activity, TPC, and amount of hydrogen peroxide was made with paired-sample *t*-test, and the IBM SPSS Statistics software (v28.0.1.1(15), New York, NY, USA) was used to calculate the *p*-values; *p* ˂ 0.05 was considered as a significant difference.

## 5. Conclusions

Red raspberry stem extracts contained varying concentrations of polyphenols that depended more on the conditions of extract preparation than on the time of plant collection. Antioxidant activity assays revealed that all stem extracts scavenged DPPH radicals, with the activity depending on quantity of polyphenols. However, TPC did not influence the hydrogen peroxide-scavenging activity. When raspberry leaf and stem extracts were prepared at temperatures below 70 °C, the scavenging of peroxide was highly active and independent of the content of polyphenols. The crucial factor for peroxide scavenging was the temperature of extract preparation. Extracts prepared at temperatures above 70 °C, infusions, and decoctions did not scavenge hydrogen peroxide, possibly due to inactivation of some enzymes involved in the reaction with peroxide. An experiment with the enzyme and polyphenol fractions of bark extract confirmed the participation of some protein (enzyme) in peroxide scavenging. Cyclic voltammetry and differential pulse voltammetry revealed the presence of easily oxidizable compounds (antioxidants) with characteristic values of oxidation potentials E_pa1_ 0.25 V and E_pa2_ 0.38 V (vs. Ag/AgCl, 3 N NaCl) as determined by differential pulse voltammetry.

Prooxidant activity as determined by hydrogen peroxide-producing activity was dependent both on total polyphenolic content and the pH of the solution.

## Figures and Tables

**Table 1:** Total polyphenolic compound content in various *R. idaeus* extracts.

Plant Organ, Harvesting Time	Extraction Conditions	TPC mg/L GA Equivalent
Stems (2018 August)	Phosph. buffer pH 6.0, room temp., 30 min.	108.91 ± 7.38 ^a^
Stems (2018 August)	Phosph. buffer pH 6.0, 30–35 °C, ultrasound, 30 min.	165.97 ± 7.31
Stems (2018 August)	Phosph. buffer pH 6.0, 50–55 °C, ultrasound, 30 min.	235.38 ± 9.88 ^b^
Stems (2018 August)	Phosph. buffer pH 6.0, 70–75 °C, ultrasound, 30 min.	333.19 ± 8.87 ^c^
Stems (2018 August)	Infusion, boiling phosph. buffer pH 6.0	254.27 ± 6.40 ^b^
Stems (2018 August)	Decoction, phosph. buffer pH 6.0, boiled for 30 min.	346.20 ± 7.58 ^c^
Stems (2018 December)	Phosph. buffer pH 6.0, 30–35 °C ultrasound, 30 min.	286.16 ± 2.94 ^b, c^
Stems (2018 December)	Infusion, phosph. buffer pH 6.0	420.00 ± 3.18
Bark (2018 December)	Phosph. buffer pH 6.0, 30–35 °C ultrasound, 30 min.	597.37 ± 8.79
Stems (2019 May)	Phosph. buffer pH 6.0, room temp., 30 min.	238.0 ± 4.23 ^b^
Leaves (2019 May)	Phosph. buffer pH 6.0, room temp., 30 min.	1290.0 ± 10.74
Bark * (for electrophoresis)		222.30 ± 5.61 ^b^
FA * fraction		115.28 ± 3.88 ^a^
FB * fraction		208.30 ± 4.88 ^b^
Stems ** (2017 December)	Phosph. buffer pH 6.0, room temp., 30 min	24.60 ± 1.43
Stems ** (2017 December)	Phosph. buffer pH 6.0, 30–35 °C, ultrasound, 30 min.	99.08 ± 3.54 ^a^

* Extract and FA, FB fractions prepared as indicated in Materials and Methods section. ** Extracts prepared after 2 years from stem harvesting. Means with no significant difference are marked with the same letters (a, b, c) (*p* > 0.05).

**Table 2:** Tentative identification of common compounds in methanolic (MeOH/H_2_O, 1:1) leaf and stem extracts of *R. idaeus* analyzed by HPLC-DAD-TOF.

Identity	t_R,_ min	Molecular Formula	Molecular Mass	Observed *m/z* [M+H]^+^, Da
Catechin (flavan-3-ol)	4.8	C_15_H_14_O_6_	290.26	291.087
Procyanidin B1(dimer of (+)-catechin and (−)-epicatechin)	6.1	C_30_H_26_O_12_	578.52	579.0692
Epicatechin	6.3	C_15_H_14_O_6_	290.26	291.0938
Gallic acid	7.9	C_7_H_6_O_5_	170.12	171.1337
Chlorogenic acid	8.1	C_16_H_18_O_9_	354.31	355.099
Coffeic acid	8.6	C_9_H_8_O_4_	180.16	181.0822
Quercetin	9.6	C_15_H_10_O_7_	302.236	303.0173
Quercitrin (quercetin-3-*O*-rhamnoside)	10.9	C_21_H_20_O_11_	448.38	449.1404
Isoquercetin (quercetin-3-*O*-glucoside)	14.1	C_21_H_20_O_12_	464.096	464.2062
Hyperoside (quercetin-3-*O*-galactoside)	15.6	C_21_H_20_O_12_	464.38	466.2202

**Table 3:** Antioxidant activity of various *R. idaeus* extracts evaluated by DPPH^●^ assay.

Extracts	DPPH^●^ Scavenging Activity TROLOX (mmol/L)
Stems (2018 December,), phosph. buffer pH 6, 30–35 °C ultrasound, 30 min	0.17 ± 0.02 ^a^
Stems (2018 December), infusion, phosph. buffer pH 6	0.38 ± 0.03
Bark (2018 December), phosph. buffer pH 6, 30–35 °C ultrasound, 30 min.	1.47 ± 0.24 ^b^
Stems (2019 May), phosph. buffer pH 6, room temp., 30 min.	1.18 ± 0.07 ^b^
Leaves (2019 May), phosph. buffer pH 6, room temp., 30 min.	1.63 ± 0.10 ^c^
Bark * (for electrophoresis)	1.90 ± 0.04
FA * fraction	0.91 ± 0.04
FB * fraction	1.66 ± 0.01 ^c^
Stems ** (2017 December), phosph. buffer pH 6, 30–35 °C, ultrasound, 30 min.	0.13 ± 0.01 ^a^

* Extract and FA, FB fractions prepared as indicated in Materials and Methods section. ** Extracts prepared after 2 years from stem harvesting. Means with no significant difference are marked with the same letters (a, b, c) (*p* > 0.05).

**Table 4:** The amount of hydrogen peroxide in *R. idaeus* stem extracts and infusions produced in 30 min.

Stem Extracts	H_2_O_2_, µmol/L
Phosph. buffer pH 4.6, 30–35 °C, ultrasound, 30 min Phosph. buffer pH 7.3, 30–35 °C, ultrasound, 30 min.	9.31 ± 0.03 14.29 ± 0.31
Infusion, boiling phosph. buffer pH 4.6, allowed to cool to room temp.	17.15 ± 0.11
Infusion, boiling phosph. buffer pH 7.3, allowed to cool to room temp.	69.81 ± 0.05

Differences in values were statistically significant (*p* ˂ 0.05).

## Data Availability

Not applicable.

## Supplementary Materials

The following supporting information can be downloaded at: https://www.mdpi.com/article/10.3390/molecules27134073/s1, Table S1. Summary of studies on bioactivities of *Rubus idaeus* leaf, stem, and rhizome extracts, Table S2. Content (g) of residuals obtained from leaves and stems of R. idaeus by decoction and extraction with different organic solvents, Table S3. Total polyphenolic content in various *R. idaeus* leaf extracts, Figure S1. UV/Vis spectra of diluted (1:50) leaf (1, 2) and stem (3, 4) R. idaeus extracts in phosphate buffer pH 6.0, Figure S2. Coomasie stained SDS-PAGE of crude *R. idaeus* plant extract and BSA standard. M—PageRuler Prestained protein ladder, FA—fraction collected after extraction and filtering of raspberry bark, FB—fraction of solution bypassed centrifugal filter, BSA—Bovine serum albumin. Bands corresponding to extracted proteins are marked by arrows, Figure S3. Gallic acid standard calibration curve, Figure S4. TROLOX standard calibration curve, Figure S5. Hydrogen peroxide standard calibration curves.

## Sample Availability

Samples of plant extracts are available from the authors.
