# Supplementary material for: In Vitro Antioxidant and Prooxidant Activities of Red Raspberry (Rubus idaeus L.) Stem Extracts
Source: Molecules. 2022 Jun 24;27(13):4073. doi: 10.3390/molecules27134073 (PMC9268408; doi:10.3390/molecules27134073)
Supplement: Supplementary file 1 [file molecules-27-04073-s001.zip › molecules-1754282-supplementary.pdf]

Table S1. Summary of studies on bioactivities of *Rubus idaeus* leaf, stem, and rhizome extracts

**Bioactivities and their research methods**	**Plant material, extractant**	**Total phenolic content (mean values)**	**Main/bioactive components**	**Evaluation of activity**	**Reference**
**Antioxidant**<br>DPPH[1]<br>phosphomolybdenum assay	Shoots, chloroform, MeOH		Ellagic acid, ellagitannins	Strong antioxidant activity.	[4]
**Antibacterial**<br>broth microdilution method				Gram-positive bacteria (β-hemolytic *Streptococcus* group A,B,G, *Streptococcus pneumoniae, Corynebacterium diphtheriae, Enterococcus faecalis, Staphylococcus aureus, Staphylococcus epidermidis, Bacillus subtilis, Clostridium sporogenes*. Gram-negative bacteria (*Klebsiella pneumoniae, Neisseria meningitidis, Moraxella catarrhalis, Haemophilus influenzae, Helicobacter pylori*), the strongest bactericidal properties against *Corynebacterium diphtheriae*.	
**Cytotoxic** (using human dermal fibroplasts, human promyelotic leukemia cell line (HL-60) and human cervical cancer cell line (HeLa)<br>MTT cell viability test				Higher cytotoxic activity towards the HL-60 cells than HeLa.	

**Antioxidant**<br>ORAC[2]	Leaves, phosphate buffer pH 7.0	47 – 129 mg GAE[3]/g of dry material, decreasing with material ageing	Not specified	High antioxidant capacities relative to those of TROLOX, α-tocopherol or vitamin C	[6]
**Relaxant** activity tested on transmurally stimulated guinea-pig ileum *in vitro*.	Leaves, solvents of different polarity (n-hexane, ethyl acetate, chloroform, MeOH)	Not specified	Not specified	The methanol extract exhibited the largest response indicating that the active compounds are of a relatively polar nature.	[7]
**Muscle relaxant** using transmurally stimulated Guinea pig ileum preparations *in vitro*.	Leaves, methanolic extract subjected to column chromatographic fractionation with chloroform-methanol mixtures	Not specified	Several triterpenoid glycosides	Triterpenoid glycosides displayed significant muscle relaxant activity.	[8]
**Antioxidant**<br>ABTS[4]<br>DPPH<br>FRAP[5]<br>**Neuroprotective**	Rhizome, 70 % EtOH subsequently partitioned by CH_2Cl_2	Not specified	Various lignans	All lignans possessed antioxidant activity.<br><br>Some lignans were effective against H_2O_2-induced SH-SY5Y human neurablastoma cell injury (in vitro).	[9]
**Antileukaemic** *in vitro* against promyelocytic HL60 cell line and its multidrug resistant sublines HL60/VINC and HL60/DOX	Leaves, acidified boiling water and thereafter MeOH added to the slurry	525 mg/100 g dry weight	Quercetin, kaempherol, ellagic, gallic, p-coumaric and caffeic acids	High cytotoxic activity.	[10]
**Radical scavenging**<br>ABTS<br>DPPH	Leaves, 96 % EtOH	4.8 to 12 mg GAE in 1 g of extract	Quercetin glucuronide, quercetin-3-O-glucoside, rutin	Effective scavengers of radicals	[11]

**Protective activity** against degradation of deoxyribose and DNA: Electrophoresis	Leaves, water infusion	Not specified	Not specified	Moderate effectiveness.	[12]
**Antioxidant**: in vitro H_2O_2-induced haemolysis assay<br><br>assay using the bacteriophage P22/*Salmonella Typhimurium* system	Leaves, water infusion	Not specified	Not specified	Effectively prevented human red blood cells from haemolysis.<br><br>High degree of protection of the bacteriophage from the oxidant challenge by H_2O_2.	[13]
**Antioxidant**: DPPH FRAP ORAC	Leaves, water (98 °C)	69 mg of GAE[9]/g of dry sample	Ellagic and gallic acids, catechin, epicatechin, procyanidin B1	About 50% of antioxidant activity of green tea extract used as a reference	[14]
**Antioxidant** DPPH ABTS<br><br>**Cytotoxic** tested using human laryngeal carcinoma (HEp2) and human adenocarcinoma of the colon (SW 480) cells	Leaves, boiled water	40.6 (mg of GAE/g of dry weight)	Flavan-3-ols, tannins, hydroxycinnamic acids, ellagic acid derivatives, quercetin derivatives	High antioxidant capacity comparable to that of green tea<br><br>Cell-type specific toxicity, more toxic to SW 480 than HEp2 after a prolonged time of exposure	[15]

**Antioxidant**<br>ORAC<br>TRAP[6]<br>HORAC[7]<br>Inhibition of lipid peroxidation<br><br>**Antimicrobial**<br>Agar diffusion assay<br><br>**Neutrophil-modulating activity**	Leaves, 80% acetone in 0.2 % formic acid	163 mg of GAE/100 g of dry weight	Epicatechin, rutin, 3,4-dihydroxybenzoic, ellagic, caffeic and gallic acids	Relatively low TRAP antioxidant activity compared to those measured by ORAC and HORAC.<br><br>Effectively inhibited the autoxidation of linoleic acid<br><br>Exhibited antimicrobial activity against *Escherichia coli*, *Salmonella* spp., *Staphylococcus aureus*, *Lysteria* spp., *Proteus vulgaris*, *Pseudomonas aruginosa*, *Klebsiella pneumoniae.*<br><br>Blocked the opsonized zymosan particle-activated ROS production by neutrophils from human whole blood.	[16]
**Antioxidant**<br>ABTS<br>DPPH<br>FRAP<br><br>**Effects** on modulation of the surface membrane expression and activity of endothelial apyrase	Leaves, 70 % acetone	270 mg GAE/g of dry extract	Ellagitannins, gallotannins	Relatively high antioxidant capacities.<br><br>Significantly augmented endothelial cell ICAM-1 expression.	[17]

(investigated by various methods)					
**Anti-inflammatory** based on human monocytic leukemia cell line THP-1 derived macrophages model	Leaves, water (40 °C)	Not specified	Ellagitannins metabolized by gut microbiota to urolithins	Extract evaluated as potential anti-inflammatory agent	[18]
**Effects** on blood platelet functions	Leaves, 70 % acetone	Not specified	Not specified.	In ADP-stimulated blood, raspberry extracts markedly decreased platelet surface membrane expression of activated GPIIbIIIa receptor and significantly inhibited platelet aggregation In platelet-rich plasma (PRP), the extract had no effect on ADP-induced platelet aggregation, modulated blood platelet reactivity in whole blood	[19]
**Antioxidant:**<br>DPPH<br>ABTS<br>FRAP<br>ORAC<br>**Cytoprotective effect** tested *in vitro* on human HepG2 cell line**:**<br>MTT[8] cell viability test	Leaves, 20 % EtOH	32 g % tannic acid	Quercitrin, isoquercitrin, quercetin, p-coumaric, caffeic and ferulic acids	Antioxidant activity was evaluated as modest compared with ascorbic and gallic acids.<br>Potential means to treat hepatic disorders associated with oxidative stress.	[20]

**Antioxidant:**<br>DPPH<br>SAS[9]<br>RP[10]<br>MCA[11]	Leaves, MeOH/water (70:30, v/v)	84 – 144 mg of GAE[9]/g of dry weight depending on the cultivar	Catechin, ellagic acid, epigallocatechin gallate, rutin	Favorable radical scavenging, capacity, adequate ferric ion reducing power, good superoxide anion radical scavenging, activity, low metal chelating ability.	[21]
**Antioxidant**<br>DPPH<br>TRAP<br>ORAC<br><br>**Antimicrobial**<br>turbidimetric assay	Leaves, acidified ethanol	228 mg GAE/100 ml of extract	Ellagitannins, flavonol glycosides	Moderate to strong (assay-dependent) antioxidant activity compared to those of tested other plant extracts<br><br>Effective against *Lysteria monocytogens*, dose-dependent activity against *E. coli*, S. aureus, *Bacilus cereus*, *Salmonella* sp.	[22]
**Antioxidant:**<br>FRAP<br>DPPH	Leaves, water (100 °C)	18 – 27 mg caffeic acid equivalents in 1 g of dry weight.	Not specified	Antioxidant activities of extracts were ranked as high and very high depending on raspberry cultivar.	[23]
**Anti-toxoplasma activity**	Aerial parts, concentrated decoction (subjected to serial extraction with various organic solvents for analysis)	Not specified	3-decen-5-one, 2-methyll (CAS),carvone, pentadecane, hexadecane	Exhibited anti-toxoplasma activity.	[24]
**Antioxidant**<br>DPPH	Leaves, MeOH	60 – 97 mg of GAE/g of extract	Flavonoids, tannins (not specified)	High level of antioxidant activity positively correlating	[25]

**Antimicrobial:** microdilution method<br><br>**Anticancer** using human colorectal cancer cell line HCT-116: cell viability and proliferation assay (MTT assay)		depending on the growth conditions		with total amount of polyphenols.<br><br>More effective against Gram-negative (*E. coli*) bacteria compared to Gram-positive (*Sarcina lutea, Bacilus subtilis*).<br><br>Negative correlation between anticancer and antioxidant activities.	
**Antioxidant** DPPH<br><br>**Antimicrobial** microwell dilution	Leaves, MeOH/acetone/water/acetic acid (30/42/27.5/0.5)	98 mg GAE/g of dry material	Quercetin glucoside, ellagic acid, rutin, kaempharol-3-glucoside, quercetin	High level of antioxidant activity.<br><br>More effective against Gram-positive (*Clostridium perfringens, Bacillus cereus, Listeria monocytogenes, Staphylococcus aureus Sarcina lutea, Micrococcus flavus*) bacteria compared to Gram-negative (*Escherichia coli, Pseudomonas aeruginosa, Salmonella enteritidis, Shigella sonnei, Klebsiella pneumoniae, Proteus*	[26]

				vulgaris) strains and yeast *Candida albicans*.	
Antioxidant<br>ABTS	Leaves, 80 % MeOH	120 – 136 mg in 100 g of fresh material depending on the cultivar (organic or conventional)	p-coumaric and ellagic acids, quercetin glucoside, quercetin, luteolin	Organic leaves were characterized by higher antioxidant activity.	[27]
**Antioxidant**<br>DPPH<br>ABTS<br>HORAC<br>FRAP<br><br>**Antidiabetic** using digestive enzymes α-glucosidase and α-amylase	Leaves, various solvents (50 % EtOH, 100 % EtOH, 50 % MeOH, 100% MeOH, ethyl acetate, acetone)	Up to 64 mg GAE/g of dry weight, depending on the solvent	Gallic and ellagic acids, procyanidin B3	The higher total phenolic amounts, the stronger antioxidant activity.<br><br>Potential inhibitors of digestive enzymes.	[28]

[1] 2,2′-diphenyl-1-picrylhydrazyl) radical.
[2] Oxygen radical absorbance capacity.
[3] Gallic acid equivalent.
[4] 2′2-azinebis-3-ethylbenzothiazolin-6-sulfonic acid.
[5] Ferric reducing antioxidant capacity.
[6] Total peroxyl-radical antioxidant parameter.
[7] Hydroxyl radical averting capacity.
[8] Dimethylthiazol-diphenyltetrazolium bromide.
[9] Superoxide anion radical scavenging activity.
[10] Reducing power.
[11] Metal chelating ability.

.

Table S2. Content (g) of residuals obtained from leaves and stems of *R. idaeus* by decoction and extraction with different organic solvents.

**Solvent**	CH_2Cl_2	**CH_3OH**	**CH_3OH/H_2O (1:1)**	**Decoction**
**Compounds**	**Nonpolar**	**Moderate polarity**	**Polar**	
Residuals from *R. idaeus* leaf extract, g	0.5805 ± 0.0910	2.0825 ± 0.2132	3.835 ± 0.3102	0.4623 ± 0.0405 [1]<br>3.5580 ± 0.2743
Residuals from *R. idaeus* stem extract, g	0.3101 ± 0.0285	1.9258 ± 0.1122	0.7631 ± 0.0930	2.2350 ± 0.1888

Decoction for 30 min,

[1] Duration of decoction procedure was 15 min

Table S3. Total polyphenolic content in various *R. idaeus* leaf extracts

Extract	Mean values of Total phenolic content, mg $GAEg^{-1}$	Country	Reference
Leaves, phosph. buff. pH 7.0, 4 °C	11 to 32 (fresh leaves), 47 to 129 (dried leaves)	USA	[6]
Leaves, 96% EtOH	4.8 to 12 (dried)	Lithuania	[11]
Leaves, hot H_2O	69 (dried)	Czech Republic	[14]
Leaves, H_2O	41 (dried)	Croatia	[15]
Leaves, 20% EtOH	32 (expressed in g% tannic acid, dried)	Romania	[20]
Leaves, MeOH:H_20 (70:30)	85 to 144 (depending on the cultivar, dried)	Serbia	[21]
Fresh leaves, EtOH/H_2O/acetic acid	2183 (100 g fresh)	Finland	[22]
Leaves, water 100 °C	18 to 27 (dried)	Poland	[23]

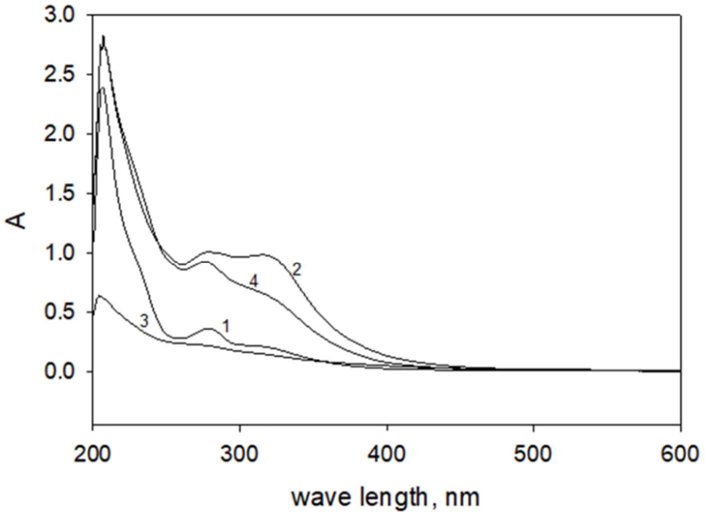

Figure S1. UV/VIS spectra of diluted (1:50) leaf (1, 2) and stem (3, 4) *R. idaeus* extracts in phosphate buffer pH 6.0.

The effect of the sample ageing is shown in Figure S1 by comparing UV/VIS spectra of extracts prepared from stems and leaves stored about a year at room temperature (Figure S1, curves 1, 3) and extracts prepared within 2 months after gathering-drying procedures (Figure S1, curves 2, 4).

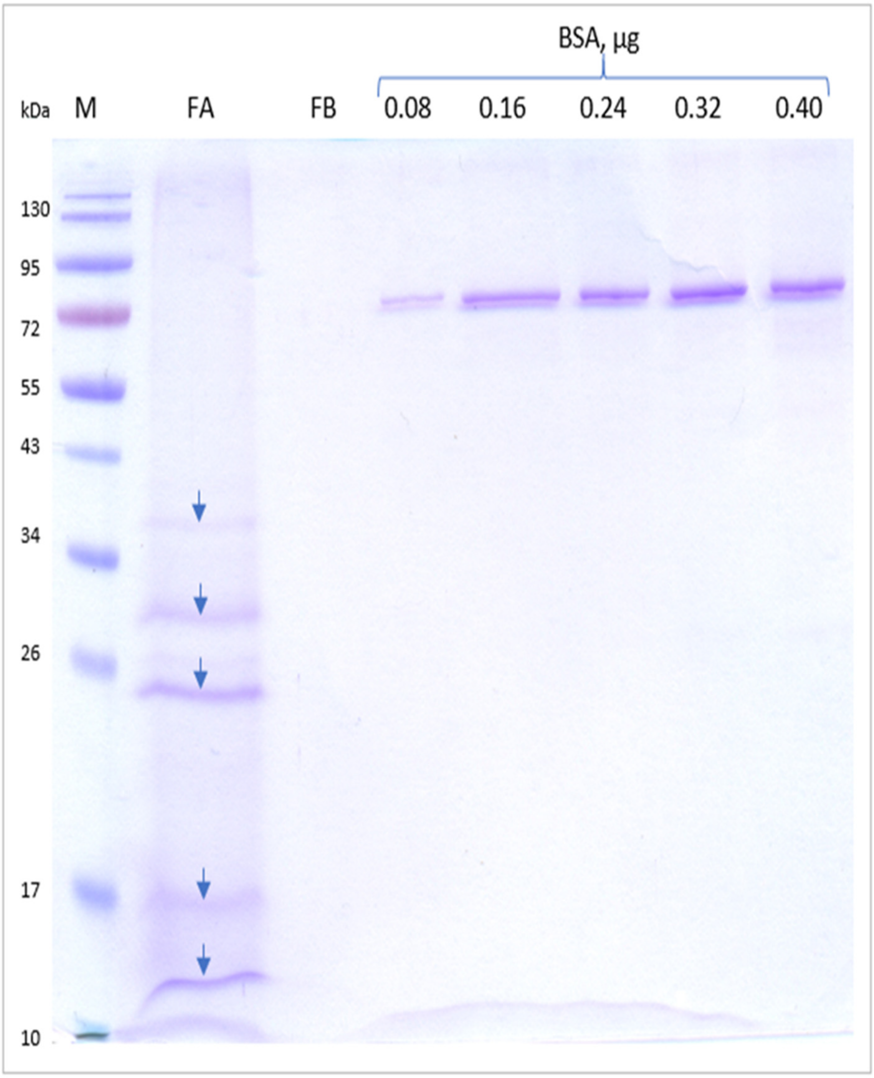

Figure S2. Coomasie stained SDS-PAGE of crude *R. idaeus* plant extract and BSA standard. M – PageRuler Prestained protein ladder, FA – fraction collected after extraction and filtering of raspberry bark, FB – fraction of solution bypassed centrifugal filter, BSA – Bovine serum albumin. Bands corresponding to extracted proteins are marked by arrows.

The amount of extracted proteins could be evaluated by visual comparison of band of unknown protein versus BSA band.

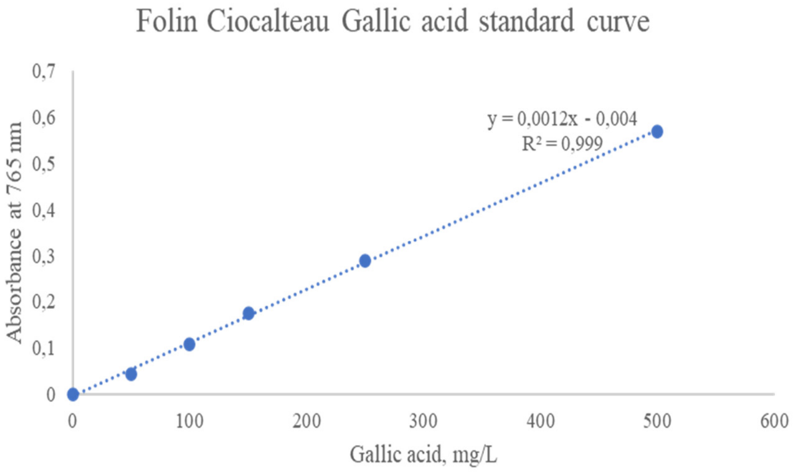

Figure S3. Gallic acid standard calibration curve.

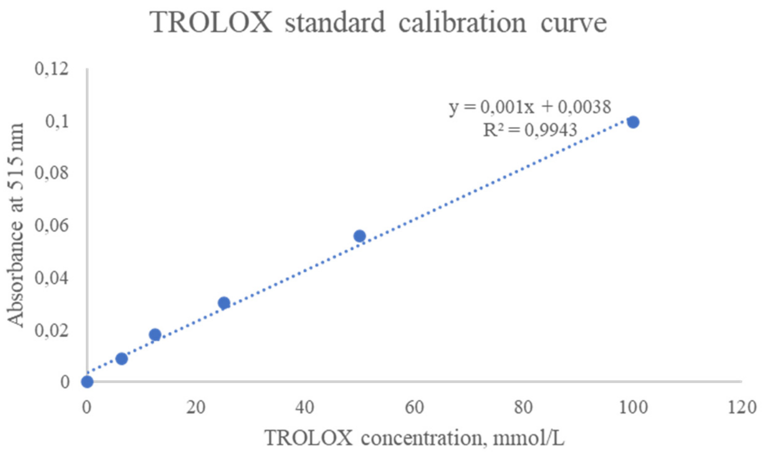

Figure S4. TROLOX standard calibration curve.

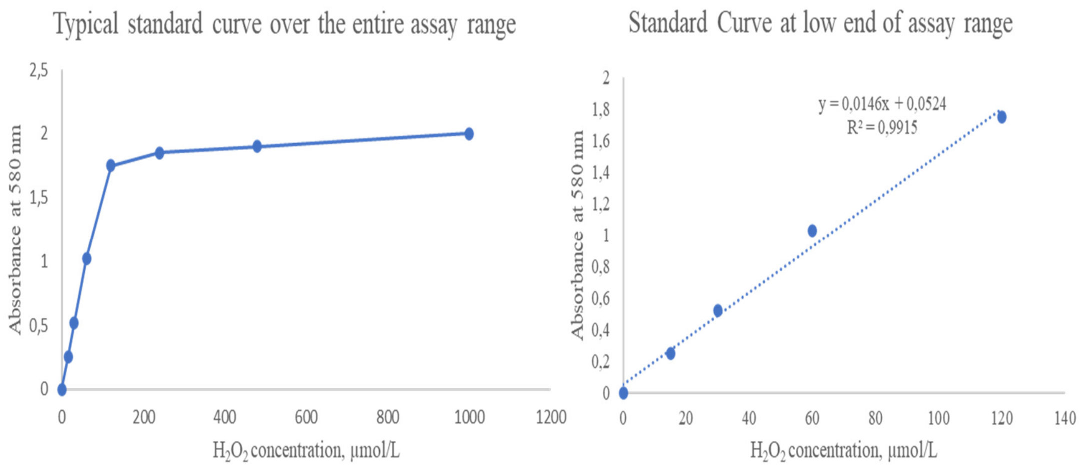

Figure S5. Hydrogen peroxide standard calibration curves.
